# The Number Needed to Treat for Music as a Medicine against Perioperative Anxiety: A Systematic Review and Meta-Analysis

**DOI:** 10.1213/ANE.0000000000007815

**Published:** 2026-03-13

**Authors:** Jetske M. Stoop, Jorrit G. Verhoeven, Sanne Hoeks, Johannes Jeekel, Markus Klimek

**Affiliations:** From the 1 Department of Neuroscience, Erasmus MC, Rotterdam, the Netherlands.; 2 Department of Anesthesiology, Erasmus MC, Rotterdam, the Netherlands.

## Abstract

**BACKGROUND:**

Music intervention is effective in reducing perioperative anxiety, which occurs in a majority of hospitalized surgical patients. A calculated Number Needed to Treat (NNT) provides an intuitive means of conveying the effectiveness of an intervention that can help clinicians decide whether or not to implement said intervention. This study aimed to calculate an NNT to provide extra context to help clinicians consider the implementation of music intervention.

**METHODS:**

To calculate the NNT of music intervention for perioperative anxiety, a systematic review and meta-analysis were performed. A comprehensive literature search was conducted in Medline ALL, Embase, Web of Science Core Collection, Cochrane, CINAHL Plus, and PsycINFO from inception until April 14, 2025. Studies describing randomized controlled trials comparing the effect of perioperative music intervention on perioperative anxiety, measured with any validated tool, were included. The revised Cochrane risk-of-bias handbook was used to determine the quality of the included studies. The NNT was calculated with Furukawa’s method, converting a calculated Cohen’s d to an NNT.

**RESULTS:**

Twenty papers were included in the review and meta-analysis. All studies used either the Visual Analog Scale for Anxiety or the 6-item State-Trait Anxiety Index. Standardized mean difference of anxiety reduction after music interventions was −0.72 (95% confidence interval [CI], −0.92 to −0.53), which equals a moderate-to-large effect size. The NNT for perioperative music intervention is 4. This indicates that 4 patients need to listen to music perioperatively, to reduce the Visual Analog Scale for Anxiety for 1 patient by 12 mm, or the State-Trait Anxiety Index by 5.7 points.

**CONCLUSIONS:**

This meta-analysis shows that a relatively low number of patients need to be treated with music intervention to reduce perioperative anxiety with an effectiveness similar to benzodiazepines.

**KEY POINTS**

- **Question:** What is the effect, and the corresponding Number Needed to Treat for music interventions to reduce anxiety in surgical patients and patients undergoing a cesarean delivery?
- **Findings:** This systematic review and meta-analysis shows that the Number Needed to Treat patients with music intervention to reduce perioperative anxiety is 4.
- **Meaning:** Music intervention is as effective as treatment with benzodiazepines without the potential negative side-effects, which should encourage clinicians to implement music intervention in daily practice.

**See Article, page 621**

Perioperative anxiety is a common occurrence in surgical patients. Prevalence studies present different results but generally show that the proportion of patients who have anxiety is >50%.^1–3^ The presence of high perioperative anxiety is not without consequences. Increased anxiety leads to increased postoperative pain,^4–6^ increased utilization of analgesics,^5–7^ reduced sleeping quality^6^ and even increased morbidity and mortality.^8–10^ There is some evidence that suggests that patients with higher levels of anxiety require more sedation to achieve adequate levels of anesthesia.^11–13^

Pharmacological treatment of perioperative anxiety has mostly revolved around the use of benzodiazepines. Nearly a quarter of patients admitted to a surgical ward in our center were treated with a benzodiazepine.^14^ However, in recent years the use of benzodiazepines as premedication has garnered scrutiny as their effectiveness seems limited.^15–17^ Moreover, side-effects such as respiratory depression, drowsiness, and dependency have led to a renewed pro-con debate about the role of benzodiazepines in perioperative care, especially in the elderly.^18^

Music intervention is a nonpharmacological alternative to treat perioperative anxiety. It is effective at reducing perioperative anxiety and relatively cheap to implement.^19–21^ In trial settings, patients are typically instructed to listen to music through head- or earphones for a given period of time. In perioperative settings, this might be done in the holding area preoperatively, intraoperatively in the operating room, or postoperatively in the nursing ward.^22^

The clinical relevance of the anxiolytic effects of music interventions is difficult to ascertain, as no minimal clinically important difference (MCID) or patient acceptable symptom state for perioperative anxiety has been established.^23^ Implementation of music interventions can therefore be challenging, and it can be helpful to have other intuitive analyses to demonstrate the effectiveness of music interventions.^24^ One such method is the calculation of a Number Needed to Treat (NNT). A calculated NNT enables comparison between the effectiveness of music interventions and medication, which can help clinicians decide on whether or not to implement said intervention.^25^ Although there have been multiple meta-analyses published on the effect of music interventions,^22,26^ none have calculated an NNT. The goal of this study is to calculate the NNT for perioperative anxiety in patients undergoing surgery using music interventions.

## METHODS

Reporting of this systematic review and meta-analysis was done in accordance with the Preferred Reporting Items for Systematic Reviews and Meta-Analyses (PRISMA) 2020 statement.^27^ The study protocol was prospectively registered in the PROSPERO database (PROSPERO no. CRD420250608218).^28^ No amendments were made after registration.

### Study Selection

The inclusion and exclusion criteria were based around the Population, Intervention, Comparison, Outcomes, and Study (PICOS) framework: (1) population: patients >16 years undergoing an intrahospital surgical procedure; (2) intervention: music interventions defined as listening to recorded music using any device from 24 hours preoperative until 7 days postoperatively, no restrictions were set on musical genre, volume or delivery method; (3) control: standard of care, meaning no intervention with the exception of head/earphones without anything playing for masking purposes; (4) outcomes: any perioperative anxiety measurement using a validated questionnaire or tool from 24 hours preoperative until 7 days postoperatively; (5) study type: randomized controlled trials.

Furthermore, articles had to be published in peer-reviewed journals, available in full-text, and published after 1990. The scope of this study was limited to papers published in English, Dutch, or German for practical and methodological reasons. These are the languages spoken and read fluently by the review team, which ensured accurate interpretation of the studies. Studies were not included if they did not provide sufficiently detailed results to be incorporated into a meta-analysis. We excluded studies that concerned minor procedures, such as dental or ophthalmological procedures, endoscopies, or outpatient surgeries. All other procedures, for example, emergency, same-day or ambulatory surgeries, were eligible for inclusion. Pilot studies and conference proceedings were excluded. Studies that examined music therapy, that is, live music therapy provided by a music therapist, were not eligible for inclusion. Two authors (J.S. and J.V.) examined the records to determine eligibility for full-text screening. Any conflicts were discussed and, if consensus could not be reached, a third, senior author (M.K.) was consulted. The full-text screening was performed in the same manner.

### Search Strategy

A comprehensive systematic literature search was made in collaboration with a biomedical information expert in electronic databases Medline ALL, Embase, Web of Science Core Collection, Cochrane, CINAHL Plus, and PsycINFO from inception until April 14, 2025 (Supplemental Digital Content 1, Supplemental File 1, https://links.lww.com/AA/F554).

### Statistical Analysis

The standardized mean difference (SMD) with 95% confidence interval (CI) was used as an effect measure, calculated as the difference between pre- to postoperative change in anxiety score in the music group, and the change in anxiety score in the control group for each study. The SMD is a summary score that is used in meta-analyses to combine the same outcomes, which are measured in different scales, into a single value.^29^ In this context, it describes the effectiveness of recorded music on anxiety. For studies with multiple intervention arms, the control group was split to equally divide the number of participants with which an intervention group was compared.^30,31^ Effect measures that were not directly reported were calculated from the available data.^32^ These were pooled in a meta-analysis using a random-effects model with the restricted maximum likelihood estimator from the metafor-package in R.^33^ Heterogeneity was assessed with the I^2^ statistic, for which a percentage of 75% or above was considered high heterogeneity.^34^ Publication bias was evaluated through a funnel plot and Egger’s test.^35^

As anxiety is commonly presented as a mean score on a continuous scale and no MCID has been established, no event rates are available, and thus, the conventional method to obtain the NNT is not possible. Strategies have been developed to calculate the NNT from Cohen’s d, among which Furukawa’s method has been proven superior as it allows for the most accurate prediction compared to other methods.^36^ Furukawa’s method requires the control event rate (CER), in this paper corresponding to the anxiety reduction in the population without music intervention.^37^ Since this has not yet been established for perioperative anxiety in the literature, an NNT was calculated for each possible CER from 0 and 1, incremented by 0.1, to provide the range the true NNT lies within. To accurately obtain the NNT, an estimate of the CER was calculated from the pooled SMD of the anxiety reduction in the control groups of the included studies. The NNT calculations were performed using the dmetar-package in R.^30^

Back transformation through the pooled standard deviation (SD) of the Visual Analog Scale for Anxiety (VAS-A) and the State-Trait Anxiety Index (STAI) was performed on the effect size to enable clinically relevant interpretation of the NNT.^38,39^

**Table 1.** Study Characteristics

Study	Country	Sample size	Surgical procedures	Age (y) Mean ± SD	Sex (%male)	Intervention	Delivery	Type of music	Control
Aker et al^22^	Turkey	80	Urological surgery	Unknown	85%	30 min preop	Headphones	Classical Turkish music	Standard care
Binns-Turner et al^23^	United States	30	Mastectomy	56.6	0%	Preop, intraop, postop at PACU	Earphones, no NC	Classical, easy listening, inspirational or new age	Earphones, no NC
Chen et al^24^	United States	70	Pelvic reconstructive surgery	60.5 (12.3)	0%	30 min preop	Headphones, NC	Instrumental	Standard care
Drzymalski et al^25^	United States	20	Cesarean delivery	36^a^	0%	Intraop	Loudspeaker	Mozart sonatas	Standard care
Drzymalski et al^26^	United States	149^d^	Cesarean delivery	34^a^	0%	Preop, intraop, 1 h postop	Loudspeaker	Mozart sonatas; Pandora radio station	Standard care
Hepp et al^27^	Germany	304	Cesarean delivery	33.6^a^	0%	Intraop	Loudspeaker	Lounge, classical, jazz or meditation	Standard care
Horasanli et al^28^	Turkey	49	Cesarean delivery	30.5^a^	0%	Intraop	Earphones	Sufi music	Standard care
Kakde et al^29^	Singapore	108	Cesarean delivery	34.0	0%	30 min preop, intraop, 30 min postop	Earphones^b^	Patient-preferred	Standard care
Kappen et al^30^	Netherlands	184	Craniotomy	60^a^	55.5%^a^	30 min preop, intraop, 2x/day 30 min until postop day 3	Headphones, no NC	Jazz, blues, classical, electronic, pop, music from different decades	Standard care
Kaur et al^31^	India	60	Cesarean delivery	26.4	0%	Intraop	Headphones	Folk, Hindi film songs or religious	Headphones
Kaur et al^32^	India	60	Cesarean delivery	Unknown	0%	Intraop	Headphones, no NC	Instrumental	Headphones, no NC
Kavak Atelma et al^33^	Turkey	117	Inguinal hernia surgery	40.2	91%	15 min preop	Headphones	Patient-preferred	Standard care
McClurkin et al^34^	United States	133^d^	Daycare surgery	54	28.6%	15 min preop; 30 min preop	Headphones	Jazz, classical, religious or natural sounds	Standard care
Nielsen et al^35^	Sweden	174	Elective awake surgery	58 (17)	53%	Intraop	Earphones	Soft instrumental music at 60-80bpm	Standard care
Nilsson et al^36^	Sweden	125	Varicose veins or open inguinal surgery	52.5	73.5%	Postop at PACU	Headphones, no NC	Soft classical music	Headphones, no NC
Nilsson et al^37^	Sweden	75^d^	Inguinal hernia surgery	57 (11.6)	96%	Intraop; 60 min postop at PACU	Headphones, NC	New-age synthesizer	Headphones, NC
Twiss et al^38^	United States	60	CABG or valve replacement surgery	73.9^a^	33%	Intraop, postop at surgical ICU	Headphones	Relaxing music, patient-preferred music after awaking	Standard care
Ugras et al^39^	Turkey	180^d^	Otorhinolaryngologic surgery^c^	35.7 (11.2)	70.6%	30 min preop	Headphones, NC	Classical Turkish music of the reed flute; classical Western music; relaxing music	Standard care
Vachiramon et al^40^	United States	100	Mohs’ micrographic surgery	64.3^a^	67%	15–60 min preop	Loudspeaker	Patient-preferred	Standard care
Wang et al^41^	China	164	Elective surgery	44.1 (10.7)	0%	30 min preop	Headphones, NC	Blues, classical, country, gospel, jazz, rhythm, Chinese popular or traditional	Headphones, NC

Abbreviations: CABG, coronary artery bypass grafting; ICU, intensive care unit; intraop, intraoperative; NC, noise-canceling; PACU, post anesthesia care unit; postop, postoperative; preop, preoperative; SD, standard deviation.

a Overall not provided by article. Given number is the average of intervention and control.

b Loudspeaker during surgery.

c Except ear surgery.

d Multiple intervention arms, comparing different types of music,^26,38^ or different timing of music,^34,36^ groups indicated with ‘;’.

**Figure 1.**
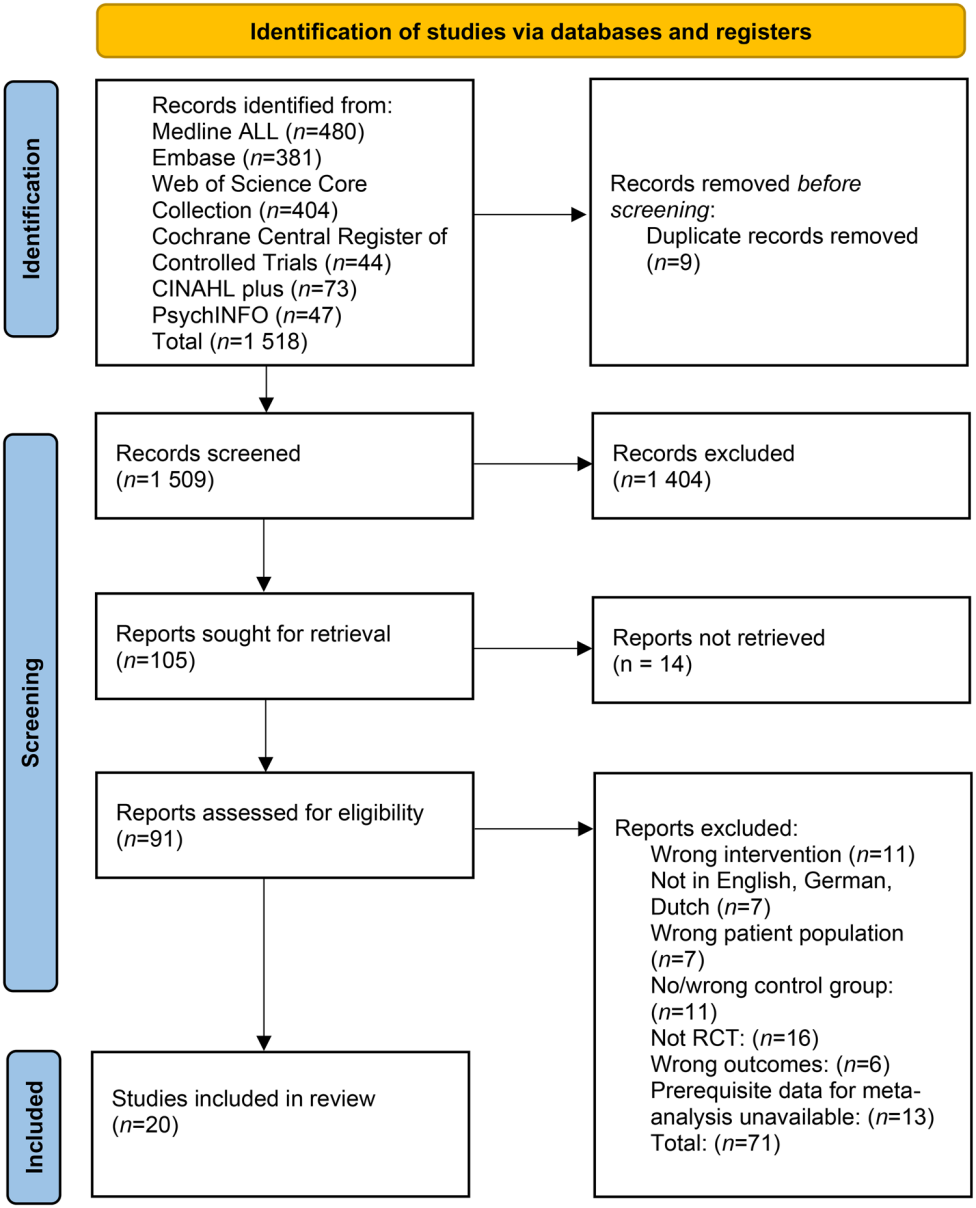
PRISMA flow chart of the study selection process. PRISMA indicates Preferred Reporting Items for Systematic Reviews and Meta-Analyses.

**Table 2.** Number Needed to Treat of Music for Perioperative Anxiety, for a Range of Control Event Rates

CER of perioperative anxiety reduction (effect size)	Number Needed to Treat
0.1	5.2
0.2	3.9
0.3	3.5
0.4	3.5
0.5	3.7
0.6	4.2
0.7	5.1
0.8	7.0
0.9	12.8

Abbreviation: CER, control event rate.

**Figure 2.**
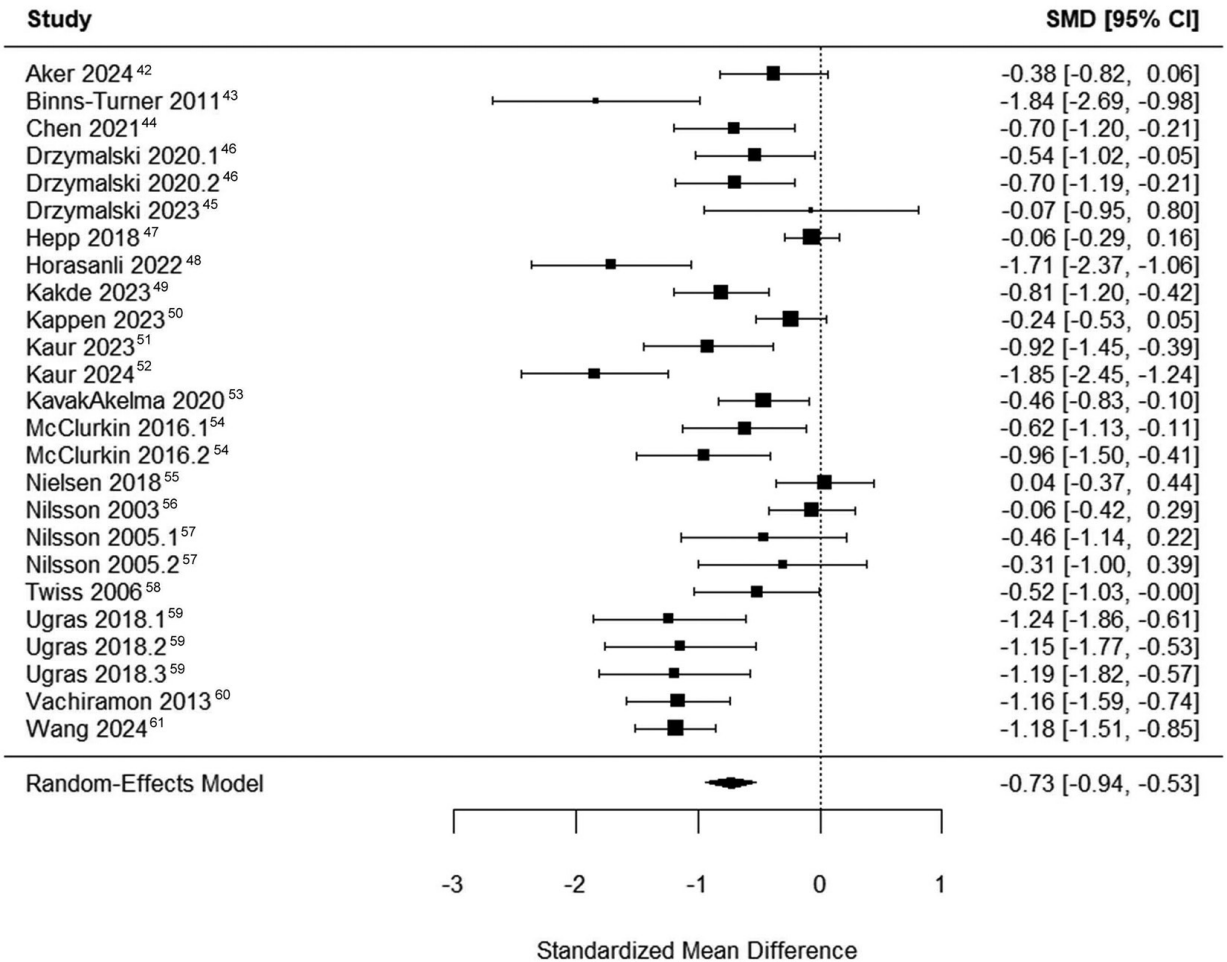
Forest plot for the effect of music on perioperative anxiety, compared with standard care. SMD indicates standardized mean difference.

To explore explanations for heterogeneity, relevant subgroup analyses were performed using similar methods as for the primary outcome on surgical procedures being cesarean sections or not, and pre- or postoperative anxiety assessment.

### Data Extraction

The following data were extracted manually from the included studies by both reviewing authors (J.S. and J.V.) using a preformatted Microsoft Excel spreadsheet and subsequently combined: author, year of publishing, country, setting, sample size, study population, surgical procedures, mean age with SD, and percentage males. Data regarding intervention and control groups were structurally extracted based on the Adapted Template for intervention Description and Replication (TiDieR) checklist for music intervention: intervention timing, frequency of sessions, time per session, delivery method, type of music, and control group.^40^ In case of missing data that were considered to be critical for the statistical analysis, the authors of the included study were contacted. For studies that partially lacked the numbers needed for the NNT calculation, all statistics were compatible to be converted into the necessary variables, and the authors of the papers did not need to be contacted to obtain the appropriate statistics for the calculation.

### Risk of Bias

Risk of bias was assessed using the revised Cochrane risk-of-bias tool for randomized controlled trials (RoB 2.0).^41^ Both reviewers (J.S. and J.V.) individually scored each study on the different domains, after which consensus was reached through discussion. After RoB 2.0, each study was labeled with an overall risk of bias based on all domains. Anxiety is a patient-reported outcome, and participants in music intervention trials are practically impossible to blind for treatment allocation; therefore, it was a priori judged to be likely that knowledge of the intervention influenced the outcome. Considering this and following the guidelines of the Cochrane Risk of Bias assessment tool, studies were expected to be graded with a high risk of bias in the domain of outcome measurement, subsequently compromising the overall score. To provide more comprehensive insights into the methodological quality of the studies, a summary score without this domain was therefore established as well.

## RESULTS

### Study Characteristics

Our initial search yielded 1427 articles after duplicate removal. Title and abstract screening, followed by full-text screening of the remaining reports, resulted in 20 articles included in the review.^42–61^ Interrater agreement of the 2 reviewers was 81%. The flow chart of the entire selection process is presented in Figure 1. The papers researched various types of surgical procedures, among which urological, gynecological, reconstructive, orthopedic, neurological, and general surgery, whereas cesarean delivery was specifically targeted in 7 studies. Music interventions were mainly applied through head- or earphones (n = 16).^42–44,48–59,61^ A wide range of genres was played. Patients could choose a preselected playlist from a number of options in 6 studies,^43,47,50,51,54,61^ and in 4 studies the music was entirely based on patient preference.^49,53,58,60^ Timing of the intervention was either preoperative (n = 7),^42,44,53,54,59–61^ intraoperative (n = 6),^45,47,48,51,52,55^ postoperative (n = 1),^56^ or throughout the perioperative period (n = 6).^43,46,49,50,57,58^ Results of the analysis of the reporting according to the TIDieR checklist are presented in Supplemental Digital Content 2, Supplemental Table 1, https://links.lww.com/AA/F555. Preexisting anxiety disorders or use of anxiolytic medication were exclusion criteria for 12 of 20 studies.^42,43,45,47,48,50–53,56,59,61^ All study characteristics are shown in Table 1.

### Number Needed to Treat

The meta-analysis was performed on all included studies, jointly involving a population of 2242 patients. The random-effects model showed an SMD of anxiety reduction after music interventions of −0.73 (95% CI, -0.94 to −0.53) (Figure 2). Subsequently, the NNT was calculated for all possible CERs, which showed that the NNT lies within a range of 3.6 and 12.9 (Table 2). The true CER was estimated at 0.20 (95% CI, −0.50 to 0.09) by pooling the SMDs from the control groups (n = 836), resulting in an NNT of music for anxiety of 3.9. Back transformation of the effect size to validated anxiety tools showed that the NNT = 3.9 clinically represents a reduction of 12 mm on the VAS-A, or 5.8 points on the STAI. A funnel plot of the results showed asymmetry, which was confirmed by Egger’s test (z = −2.56, *P* = .01) (Supplemental Digital Content 3, Supplemental Figure 1, https://links.lww.com/AA/F556).

### Subgroup Analysis

The primary random-effects model showed high levels of heterogeneity. To explore potential explanations, subgroup analyses were performed on the type of surgery and on the timing of anxiety assessment.

As a large proportion of the included studies targeted pregnant women undergoing surgical delivery (n = 7),^45–49,51,52^ cesarean sections formed a subgroup, compared with other surgeries (n = 13).^42–44,50,53–61^ Meta-analysis of cesarean sections (n = 750), versus other surgeries (n = 1 492), showed an SMD of −0.82 (95% CI, -1.27 to -0.38) and −0.69 (95% CI, −0.92 to −0.46), respectively (Supplemental Digital Content 4, Supplemental Figures 2.1 and 2.2, https://links.lww.com/AA/F557). The NNT was 3.2 and 4.4, respectively.

**Figure 3.**
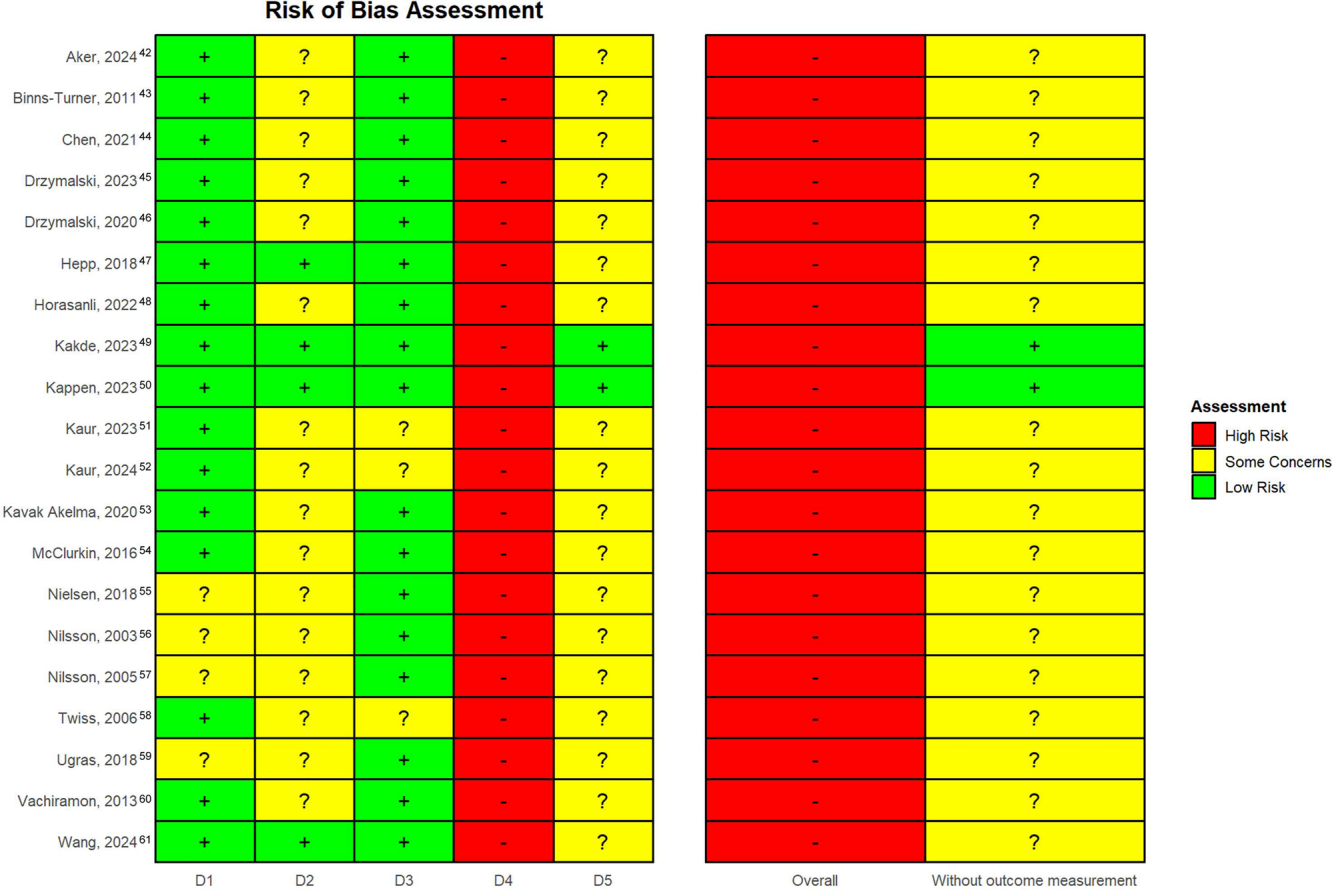
Risk of bias assessment. D1 indicates randomization; D2, intervention assignment; D3, missing data; D4, outcome measurement; D5, selection of reported results.

In 6 papers preoperative anxiety was the outcome of interest,^42,44,53,54,59,61^ and 14 papers compared anxiety reduction in the intervention and control groups postoperatively,^43,45–52,55–58,60^ whereas all studies measured baseline anxiety preoperatively (Supplemental Digital Content 5, Supplemental Table 2, https://links.lww.com/AA/F558). Therefore, subgroup analyses for the timing of anxiety assessment were performed and showed an SMD of −0.91 (95% CI, −1.15 to −0.67) for preoperative anxiety (n = 744), and −0.65 (95% CI, −0.93 to −0.38) for postoperative anxiety (n = 1498) (Supplemental Digital Content 6, Supplemental Figures 3.1 and 3.2, https://links.lww.com/AA/F559). The NNT was 21.9 and 4.0, respectively.

### Risk of Bias

In accordance with prior expectations, all studies were rated with low overall quality due to poor scoring in the outcome assessment domain (Figure 3). The summary score without this domain shows moderate quality of most studies, mainly due to issues regarding the domain on intervention assignment as well as selection of the reported results. The studies with some concerns did not report any information on adherence to the intervention, or had not published a protocol, providing transparency through a prespecified analysis plan, respectively. Within the domains of randomization or missing data, most studies were evaluated with a low risk of bias.

## DISCUSSION

This study set out to calculate an NNT for music intervention of anxiety, one of the most common perioperative complications. We found that music intervention reduces perioperative anxiety significantly with a moderate-to-large effect size. Our results show that the NNT for perioperative anxiety reduction using music intervention is 4. To elaborate and explain, when 4 patients are treated with a perioperative music intervention, the VAS-A for 1 patient is reduced by 12 mm. For the STAI score, this corresponds to a 5.8 point reduction in STAI score on a scale from 20 to 80. To the best of our knowledge, this is the first published NNT for music interventions, which is in line with the use of “music as a medicine.”

The interpretation of this NNT remains challenging for clinicians who are not used to working with the VAS-A or STAI scores regularly. However, the STAI-6 and VAS-A are commonly used in previous meta-analyses and studies investigating music interventions. Other than for perioperative pain, an MCID for perioperative anxiety has not been established.^62^ In the literature, a universally accepted MCID, when no studies have been performed to calculate an outcome-specific clinically relevant difference, in our case anxiety, is an SD of 0.5 of the used scale, which translates to a 10 mm reduction on the VAS-A scale.^63^ By comparison, a study examining the MCID for anxiety in patients with chronic obstructive pulmonary disease established a difference of 1.32 in the Hospital Anxiety and Depression Scale-Anxiety (HADS-Anxiety).^64^ A similar study comparing the effect of rehabilitation on patients with severe bronchiectasis reported an MCID of 2 for HADS-Anxiety.^65^ A study on the effect of rotator cuff surgery on anxiety and depression described an MCID of 3.8 for HADS-Anxiety in that population.^66^ We caution against using these MCIDs for perioperative anxiety because of the differences in study populations. For the sake of illustration, on average, after transformation to VAS-A and STAI-6, the MCID for anxiety in these studies corresponds to an 11.3 mm and a 9-point difference, respectively.

Another way to try to interpret our results would be to compare established NNTs of treatments that aim to achieve a similar effect. Benzodiazepines are often used as premedication in anesthesiologic procedures before surgery for their anxiolytic effects, although their effectiveness in improving postoperative recovery is debatable.^15–17^ The only NNT for the use of benzodiazepines is in the context of treatment of a panic disorder, in which the NNT is 4.^67^ In any case, no NNT for the effect of benzodiazepines on preoperative anxiety is known, limiting our ability to make a direct comparison with the nonpharmacological alternative, music intervention. Nevertheless, we would argue it makes the case for the use of music intervention as it is a safe (no known side effects, no risk of dependency), cheap^40,68^ and effective treatment for not only perioperative anxiety but also pain, stress, sleep disorders, and delirium.^21,24,69–71^

With regard to the secondary analyses, cesarean sections were assessed in a subgroup to compare with other surgical procedures. The prognosis and emotional impact naturally are different for surgeries for disease treatment than for childbirth, and, whilst women do not automatically have more anxiety perioperatively, they usually do report a higher level than men.^2^ Supporting the fact that we found no significant difference between the SMD or the NNT of cesarean sections or other types of surgical procedures, all C-sections were planned for medical indications, which aligns with the elective nature of the procedures that were investigated in the noncesarean delivery studies. The preclusion of most anxiolytic medications for these procedures is not likely to have affected the results, as most other studies also excluded patients with preexisting anxiety or anxiolytic use. In contrast, we found that the NNT to reduce preoperative anxiety was 5 times larger than the NNT to reduce anxiety during surgery until postoperatively. From an anesthesiologic perspective, the preoperative NNT is clinically relevant as preoperative anxiety is the area over which anesthesiologists have the most influence. Besides, it forms a risk factor for postoperative anxiety as well.^72^ Yet, since treatment of preoperative anxiety is intended to improve the status and recovery of the patient postoperatively, we reckon the postoperative effects and NNT of music intervention to be as relevant.^15–17^ This higher NNT for preoperative anxiety should be interpreted with caution, since we found a lower baseline anxiety in the studies that investigated preoperative anxiety. We hypothesize this is due to the fact that these studies included same-day surgery and otorhinolaryngology surgery patients, who might have lower baseline anxiety when compared with patients undergoing thoracic or abdominal oncological procedures. Therefore, the CER is likely to be smaller, which could affect the NNT substantially, as will be explained below in the limitations of Furukawa’s method. Another potential explanation is that the sample size of this subgroup was relatively small, and the concerning studies were the ones with the lowest methodological quality and could not accurately capture the anxiety levels.^42,44,53,54,59,61^

We like to point out that live music therapy was not included in this review. Whilst there are clear indications of the effectiveness, the intervention is too different from the recorded music intervention to be able to compare the results of these studies. Live music therapy involves a trained music therapist, who engages with the patient and therefore, introduces a relational component; it is costly and less feasible to implement in standard care for many patients at once.^73^

### Limitations and Strengths

A limitation of our study is the low quality of evidence of the individual studies and the moderate-to-high risk of bias, which is partially explained by the nature of the intervention. The lack of blinding could have influenced the reported outcomes and could have led to either an under- or overestimation of the effect of music. Furthermore, there is heterogeneity in the timing of the music intervention, music genre, and delivery method, the study populations, and the surgical procedures, which challenges the reproducibility and generalizability of our findings. Our results also indicate signs of publication bias of positive results, possibly leading to an overestimation of the results. Lastly, the Furukawa method requires an estimation of the CER to enable the calculation of an NNT for a continuous outcome such as anxiety. Though this is the feature that allows for accurate prediction of the NNT, small changes in this CER may have substantial differences in the calculated NNT. The absolute benefit of a treatment, and thus the NNT, is maximized when the CER is moderate. The benefit will become smaller, which minimizes the NNT, when the CER is very low or very high. This remains true even if the effect size remains constant, as was visible in the values of the NNT for a range of CERs.^36^

Strengths of our study are the strict inclusion criteria used and the comprehensiveness of our search strategy. There is a lot of research on music, and we have strived to only include studies from a certain standard, quality-wise.

## CONCLUSIONS

The NNT patients with music intervention to reduce perioperative anxiety is 4. The interpretation is complicated by the lack of a defined minimal clinically important difference for anxiety and of a comparable NNT for similar interventions. However, it is clear from this meta-analysis that music intervention is effective at reducing perioperative anxiety in a relatively low number of treated patients. This should encourage clinicians to implement music intervention in daily practice.

## ACKNOWLEDGMENTS

The authors wish to thank Dr Wichor Bramer, PhD, from the Erasmus MC Medical Library (Rotterdam, the Netherlands) for developing and updating the search strategies. Author Prof Dr Johannes J (Hans) Jeekel sadly passed away during the revisions for this manuscript. We sincerely hope that the publication of this piece honors his legacy and that he would have been proud of the final result.

## DISCLOSURES

**Conflicts of Interest:** None. **Funding:** This work was supported by the Erasmus MC Foundation. **This manuscript was handled by:** Girish P. Joshi, MBBS, MD, FFARCSI.

## Supplementary Material


