# Supplementary material for: The Number Needed to Treat for Music as a Medicine against Perioperative Anxiety: A Systematic Review and Meta-Analysis
Source: Anesth Analg. 2026 Mar 13;142(4):625–34. doi: 10.1213/ANE.0000000000007815 (PMC12959583; doi:10.1213/ANE.0000000000007815)
Supplement: Supplementary file 1 [file ane-142-625-s001.pdf]

Supplemental File 1. Search strategy.

**Database searched**	**Platform**	**Years of coverage**	**Records**	**Records after duplicates removed**
Medline ALL	Ovid	1946 - Present	510	508
Embase	Embase.com	1971 - Present	855	415
Web of Science Core Collection*	Web of Knowledge	1975 - Present	842	424
Cochrane Central Register of Controlled Trials	Wiley	1992 - Present	311	44
CINAHL Plus	EBSCO	1982 - Present	308	79
PsycINFO	Ovid	1806 - Present	134	48
**Total**			**2960**	**1518**

*Science Citation Index Expanded (1975-present) ; Social Sciences Citation Index (1975-present) ; Arts & Humanities Citation Index (1975-present) ; Conference Proceedings Citation Index- Science (1990-present) ; Conference Proceedings Citation Index- Social Science & Humanities (1990-present) ; Emerging Sources Citation Index (2005-present)

No other database limits were used than those specified in the search strategies

medline
(Music / OR (music OR musical OR musicotherap*).ab,ti.) AND (Surgical Procedures, Operative/ OR exp Obstetric Surgical Procedures/ OR Postoperative Complications/ OR Perioperative Nursing/ OR Postanesthesia Nursing/ OR Operating Rooms/ OR Recovery Room/ OR (surger* OR surgic* OR peroperat* OR perioperat* OR preoperat* OR postoperat* OR operati* OR interoperat* OR intraoperat* OR anesthe* OR anaesthe* OR perianesthe* OR peranesthe* OR perianaesthe* OR peranaesthe* OR preanasthe* OR preanaesthe* OR postanasthe* OR postanaesthe*).ab,ti. OR surgery.fx.) AND (Anxiety/ OR Fear/ OR Anxiety Disorders/ OR (anxiet* OR anxious* OR fear* OR vas-a OR stai).ab,ti,kw.) NOT (music-therap*).ti. NOT (exp animals/ NOT humans/)

Embase
(music/de OR (music OR musical OR musicotherap*):ab,ti) AND (surgery/exp OR 'obstetric operation'/exp OR 'postoperative complication'/exp OR 'anesthesiological procedure'/exp OR 'perioperative nursing'/de OR 'postanesthesia nursing'/de OR 'operating room'/de OR 'recovery room'/de OR 'operating room personnel'/de OR (surger* OR surgic* OR peroperat* OR perioperat* OR preoperat* OR postoperat* OR operati* OR interoperat* OR intraoperat* OR anesthe* OR anaesthe* OR perianesthe* OR peranesthe* OR perianaesthe* OR peranaesthe* OR preanasthe* OR preanaesthe* OR postanasthe* OR postanaesthe*):ab,ti OR surgery:lnk) AND (anxiety/de OR 'anticipatory anxiety'/de OR 'fear of pain'/de OR 'fear of death'/de OR fear/de OR 'anxiety assessment'/exp OR 'anxiety disorder'/de OR (anxiet* OR anxious* OR fear* OR vas-a OR stai):ab,ti,kw) NOT (music-therap*):ti NOT [conference abstract]/lim NOT ([animals]/lim NOT [humans]/lim)

Web of science
TS=((music OR musical OR musicotherap*)) AND TS=((surger* OR surgic* OR peroperat* OR perioperat* OR preoperat* OR postoperat* OR operati* OR interoperat* OR intraoperat* OR anesthe* OR anaesthe* OR perianesthe* OR peranesthe* OR perianaesthe* OR peranaesthe* OR preanasthe* OR preanaesthe* OR postanasthe* OR postanaesthe*)) AND (TS=(anxiet* OR anxious* OR fear* OR vas-a OR stai)) NOT TI=(music-therap*) NOT DT=(Meeting Abstract OR Meeting Summary) AND LA=(English)

Cochrane

((music OR musical OR musicotherap*):ab,ti) AND ((surger* OR surgic* OR peroperat* OR perioperat* OR preoperat* OR postoperat* OR operati* OR interoperat* OR intraoperat* OR anesthe* OR anaesthe* OR perianesthe* OR peranesthe* OR perianaesthe* OR peranaesthe* OR preanasthe* OR preanaesthe* OR postanasthe* OR postanaesthe*):ab,ti) AND ((anxiet* OR anxious* OR fear* OR vas-a OR stai):ab,ti,kw)
(music-therap*):ti
("conference abstract":kw OR Trial registry record:pt)
#1 NOT #2 NOT #3

PsycINFO

(Music / OR (music OR musical OR musicotherap*).ab,ti.) AND (Surgery/ OR Postsurgical Complications / OR (surger* OR surgic* OR peroperat* OR perioperat* OR preoperat* OR postoperat* OR operati* OR interoperat* OR intraoperat* OR anesthe* OR anaesthe* OR perianesthe* OR peranesthe* OR perianaesthe* OR peranaesthe* OR preanasthe* OR preanaesthe* OR postanasthe* OR postanaesthe*).ab,ti.) AND (Anxiety/ OR Fear/ OR Anxiety Disorders/ OR (anxiet* OR anxious* OR fear* OR vas-a OR stai).ab,ti.) NOT (music-therap*).ti. NOT (exp animals/ NOT humans/)

CINAHL

(MH Music + OR TI(music OR musical OR musicotherap*) OR AB(music OR musical OR musicotherap*)) AND (Surgery, Operative+ OR MH Postoperative Complications+ OR MH Perioperative Nursing OR MH Perianesthesia Nursing OR MH Operating Rooms+ OR TI(surger* OR surgic* OR peroperat* OR perioperat* OR preoperat* OR postoperat* OR operati* OR interoperat* OR intraoperat* OR anesthe* OR anaesthe* OR perianesthe* OR peranesthe* OR perianaesthe* OR peranaesthe* OR preanasthe* OR preanaesthe* OR postanasthe* OR postanaesthe*) OR AB(surger* OR surgic* OR peroperat* OR perioperat* OR preoperat* OR postoperat* OR operati* OR interoperat* OR intraoperat* OR anesthe* OR anaesthe* OR perianesthe* OR peranesthe* OR perianaesthe* OR peranaesthe* OR preanasthe* OR preanaesthe* OR postanasthe* OR postanaesthe*)) AND (MH Anxiety OR MH Fear OR MH Anxiety Disorders OR TI(anxiet* OR anxious* OR fear* OR vas-a OR stai) OR AB(anxiet* OR anxious* OR fear* OR vas-a OR stai)) NOT TI(music-therap*) NOT (MH animals+ NOT MH humans+)
