# Supplementary material for: The Number Needed to Treat for Music as a Medicine against Perioperative Anxiety: A Systematic Review and Meta-Analysis
Source: Anesth Analg. 2026 Mar 13;142(4):625–34. doi: 10.1213/ANE.0000000000007815 (PMC12959583; doi:10.1213/ANE.0000000000007815)
Supplement: Supplementary file 2 [file ane-142-625-s002.pdf]

Study	1	2	3a	3b	3c	3d	3e	4a	4b	5	6	7	8a	8b	8c	9	10	11[c]	12[c]
**Aker et al., 2024[42]**	+	+	+	?[a]	+	-	-	+	+	-	+	+	+	+	+	NA	NA	?	?
**Binns-Turner et al., 2011[43]**	+	+	+	+	+	-	+	+	+	-	+	+	+	+	?	NA	NA	?	?
**Chen et al., 2021[44]**	+	+	+	+	+	-	-	+	+	-	+	+	+	+	+	NA	NA	?	?
**Drzymalski et al., 2023[45]**	+	+	+	+	+	+	-	+	+	-	+	+	+	+	?[b]	NA	NA	?	?
**Drzymalski et al., 2020[46]**	+	+	+	+	+	+	-	+	+	-	+	+	+	+	?[b]	NA	NA	?	?
**Hepp et al., 2018[47]**	+	+	+	+	+	-	-	+	+	-	+	+	+	+	+	NA	NA	+	+
**Horasanli et al., 2022[48]**	+	+	-	+	+	+	+	+	+	-	+	+	+	+	?[b]	NA	NA	?	?
**Kakde et al., 2023[49]**	+	+	+	?[a]	+	+	-	+	+	+	+	+	+	+	+	NA	NA	+	+
**Kappen et al., 2023[50]**	+	+	+	+	+	+	-	+	+	+	+	+	+	+	+	NA	NA	+	+
**Kaur et al., 2023[51]**	+	+	+	?[a]	+	-	-	+	+	-	+	+	+	+	?[b]	NA	NA	?	?
**Kaur et al., 2024[52]**	+	+	+	+	-	-	+	+	+	+	+	+	+	+	?[b]	NA	NA	?	?
**Kavak Akelma et al., 2020[53]**	+	+	-	?[a]	+	+	-	+	+	-	+	+	+	+	+	NA	NA	?	?
**McClurkin et al., 2016[54]**	+	+	+	?[a]	+	-	-	+	+	+	+	+	+	+	+	NA	NA	?	?
**Nielsen et al., 2018[55]**	+	+	+	?[a]	+	+	-	+	+	+	+	+	+	+	?[b]	NA	NA	?	?
**Nilsson et al., 2003[56]**	+	+	-	+	+	+	-	+	+	-	+	+	+	+	?	NA	NA	?	?
**Nilsson et al., 2005[57]**	+	+	+	+	+	-	-	+	+	+	+	+	+	+	+	NA	NA	?	?
**Twiss et al., 2006[58]**	+	+	+	?[a]	+	+	-	+	+	+	+	+	+	+	?[b]	NA	NA	?	?
**Ugras et al., 2018[59]**	+	+	+	+	+	+	-	+	+	-	+	+	+	+	+	NA	NA	?	?
**Vachiramon et al., 2013[60]**	+	+	+	+	+	+	-	+	+	+	+	+	+	+	+	NA	NA	?	?
**Wang et al., 2024[61]**	+	+	+	+	+	-	-	+	+	+	+	+	+	+	+	NA	NA	+	+

Supplemental Table 1. Reporting of the music intervention according to the TIDieR checklist.

1 = Brief name, 2 = Why, 3a = Audio player, 3b = Music output device, 3c = Music content, 3d = Music selection, 3e = Information, 4a = Intervention group, 4b = Control group, 5 = Who provided, 6 = How, 7 = Where, 8a = Number of days, 8b = Frequency, 8c = Duration, 9 = Tailoring, 10 = Modifications, 11 = How well: Planned, 12 = How well: Actual.

[a] *Unknown whether ear- or headphones were noise-cancelling*

[b] *Intraoperative intervention; duration of surgery not specified, thus exact duration of intervention unknown.*

[c] *'?' in case adherence to the intervention was not assessed*
