# Supplementary figures and images for: The Number Needed to Treat for Music as a Medicine against Perioperative Anxiety: A Systematic Review and Meta-Analysis
Source: Anesth Analg. 2026 Mar 13;142(4):625–34. doi: 10.1213/ANE.0000000000007815 (PMC12959583; doi:10.1213/ANE.0000000000007815)

**Supplemental Figure 1.** Funnel plot of the effect of music on perioperative anxiety.

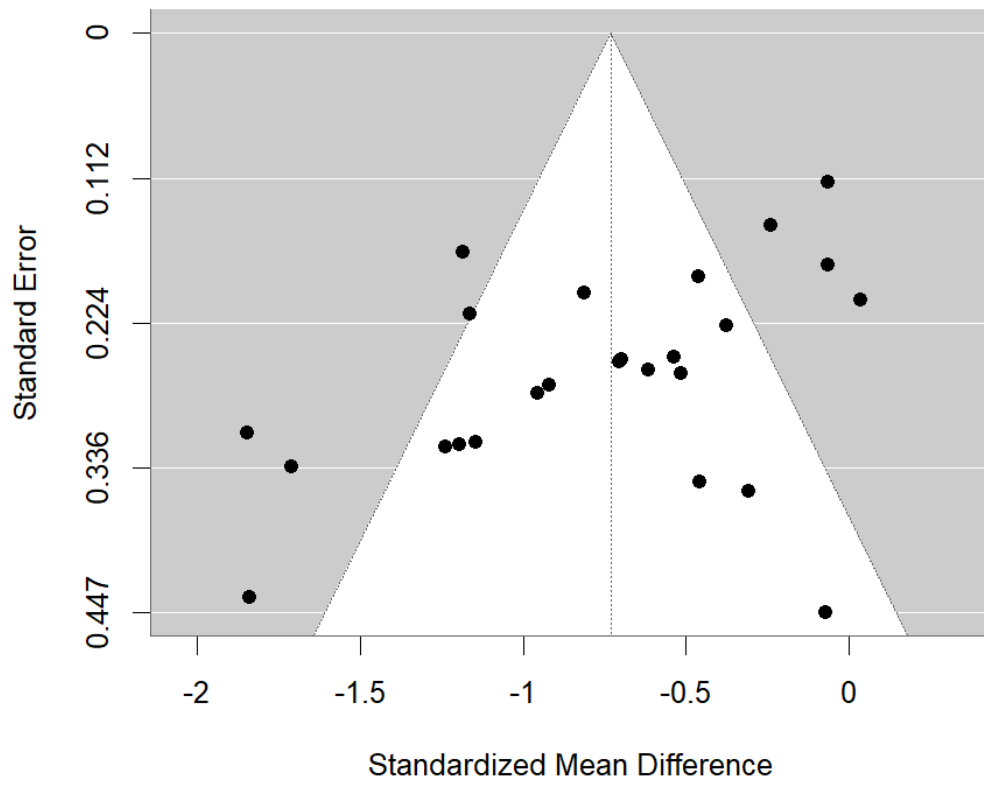

Supplement: Supplementary file 3 [file ane-142-625-s003.pdf]
