# Supplementary material for: The Number Needed to Treat for Music as a Medicine against Perioperative Anxiety: A Systematic Review and Meta-Analysis
Source: Anesth Analg. 2026 Mar 13;142(4):625–34. doi: 10.1213/ANE.0000000000007815 (PMC12959583; doi:10.1213/ANE.0000000000007815)

Supplemental Figure 2.1. Forest plot for the effect of music on perioperative anxiety, compared with standard care for cesarean sections.
SMD = standardized mean difference.

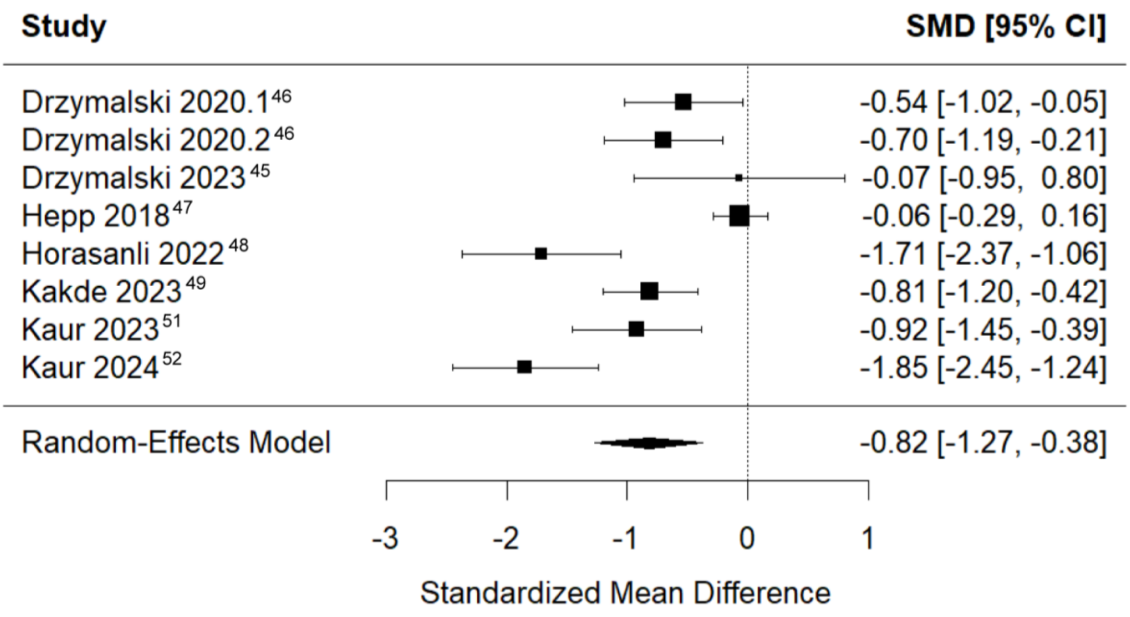

Supplemental Figure 2.2. Forest plot for the effect of music on perioperative anxiety, compared with standard care for all other surgeries.
SMD = standardized mean difference.

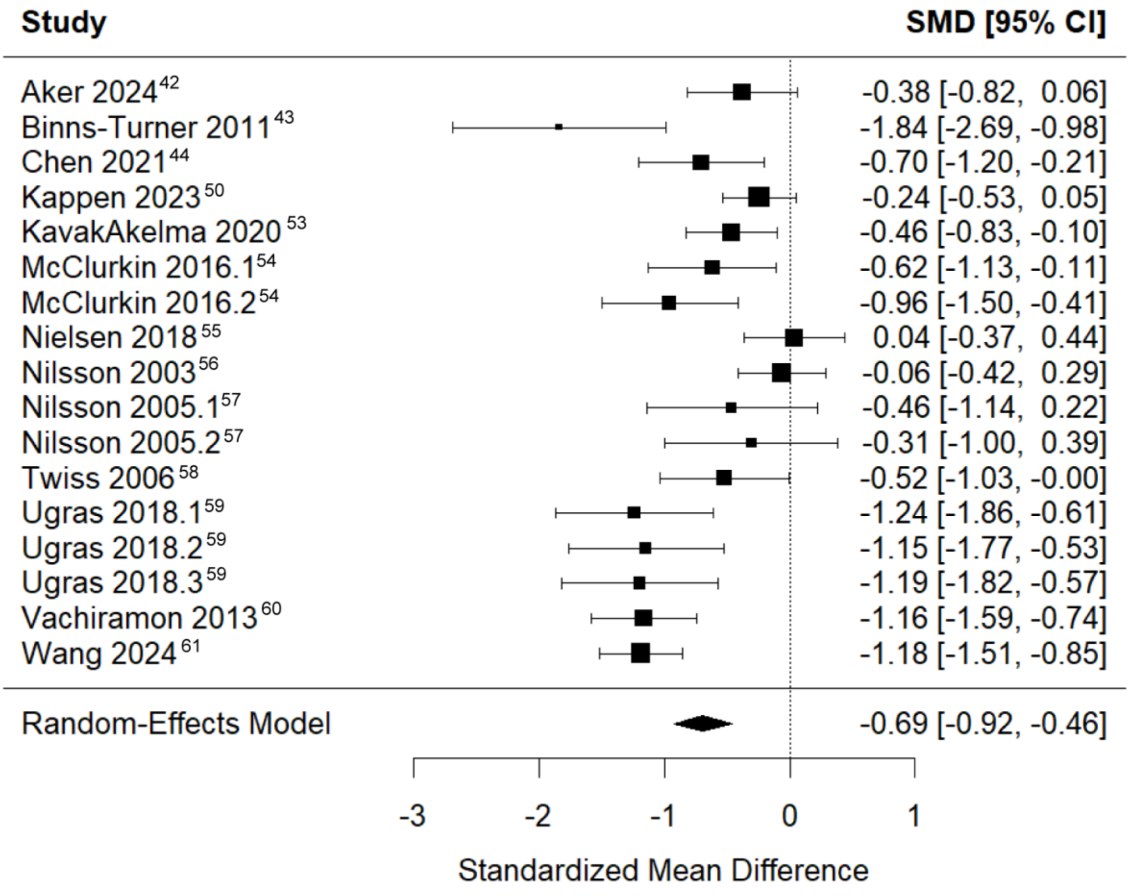

Supplement: Supplementary file 4 [file ane-142-625-s004.pdf]
