# Supplementary material for: The Number Needed to Treat for Music as a Medicine against Perioperative Anxiety: A Systematic Review and Meta-Analysis
Source: Anesth Analg. 2026 Mar 13;142(4):625–34. doi: 10.1213/ANE.0000000000007815 (PMC12959583; doi:10.1213/ANE.0000000000007815)
Supplement: Supplementary file 5 [file ane-142-625-s005.pdf]

Study	Sample size	Timing measurement	Anxiety assessment tool	Mean ± SD, change in anxiety, intervention	Mean ± SD, change in anxiety, control
Aker et al., 2024[42]	80	Preoperative	STAI	-2.48 ± 8.61	1 ± 9.61
Binns-Turner et al., 2011[43]	30	Pre and postoperative	STAI	-10.8 ± 7.7	7.8 ± 11.6
Chen et al., 2021[44]	70	Pre-operative	STAI	-6.69 ± 6.98	-1.32 ± 8.03
Drzymalski et al., 2023[45]	20	Pre and postoperative	NRS	-2.7 ± 2.7	-2.5 ± 2.6
Drzymalski et al., 2020[46]	149	Pre and postoperative	NRS	1.3 ± 0.8<br>1.4 ± 1	2 ± 1.3
Hepp et al., 2018[47]	304	Pre and postoperative	STAI, VAS-A	-18 ± 9.03	-17.4 ± 10.3
Horasanli et al., 2022[48]	49	Pre and postoperative	STAI	-9.7 ± 5.04	-1.68 ± 4.07
Kakde et al., 2023[49]	108	Pre and postoperative	VAS-A	-3.7 ± 2.14	-1.8 ± 2.51
Kappen et al., 2023[50]	184	Pre and postoperative	VAS-A	-0.25 ± 1.49	0.05 ± 0.94
Kaur et al., 2023[51]	60	Pre and postoperative	VAS-A	-0.87 ± 0.70	-0.11 ± 0.92
Kaur et al., 2024[52]	60	Pre and postoperative	VAS-A	-5.1 ± 1.47	-2.07 ± 1.76
Kavak Akelma et al., 2020[53]	117	Pre-operative	STAI	-6.71 ± 6.89	-3.98 ± 4.63
McClurkin et al., 2016[54]	133	Pre-operative	STAI, VAS-A	-7.1 ± 9.8<br>-9.2 ± 8.44	-1.5 ± 6.93
Nielsen et al., 2018[55]	174	Pre and postoperative	STAI, VAS-A	3 ± 10.8	2.67 ± 5.13
Nilsson et al., 2003[56]	125	Pre and postoperative	STAI	-4.8 ± 7.95	-4.3 ± 7.35
Nilsson et al., 2005[57]	75	Pre and postoperative	NRS	-2.1 ± 2.19<br>-1.7 ± 1.87	-1.1 ± 2.0
Twiss et al., 2006[58]	60	Pre and postoperative	STAI	-7.67 ± 10.6	-1.76 ± 12.0
Ugras et al., 2018[59]	180	Pre-operative	STAI	-4.73 ± 3.21<br>-6.22 ± 5.63<br>-6.27 ± 5.37	0.56 ± 6.44
Vachiramon et al., 2013[60]	100	Pre and postoperative	STAI, VAS-A	-9.9 ± 7.4	-3.4 ± 2.6
Wang et al., 2024[61]	164	Pre-operative	STAI	-7.7 ± 7.4	0.7 ± 6.7

Supplemental Table 2. Change in anxiety scores before and after intervention.
STAI = State-Trait Anxiety Index, NRS = Numeric Rating Scale, VAS-A = Visual Analogue Scale for Anxiety.
